# The Contribution of Different Components in QiShenYiQi Pills® to Its Potential to Modulate Energy Metabolism in Protection of Ischemic Myocardial Injury

**DOI:** 10.3389/fphys.2018.00389

**Published:** 2018-04-11

**Authors:** Yuan-Chen Cui, Li Yan, Chun-Shui Pan, Bai-He Hu, Xin Chang, Jing-Yu Fan, Jing-Yan Han

**Affiliations:** ^1^Tasly Microcirculation Research Center, Peking University Health Science Center, Beijing, China; ^2^Key Laboratory of Microcirculation, State Administration of Traditional Chinese Medicine of the People's Republic of China, Beijing, China; ^3^Key Laboratory of Stasis and Phlegm, State Administration of Traditional Chinese Medicine of the People's Republic of China, Beijing, China; ^4^Beijing Laboratory of Integrative Microangiopathy, Beijing, China; ^5^State Key Laboratory of Core Technology in Innovative Chinese Medicine, Beijing, China; ^6^Department of Integration of Chinese and Western Medicine, School of Basic Medical Sciences, Peking University, Beijing, China

**Keywords:** ischemic heart disease, ATP synthesis, cardiac structure, F-actin, energy metabolism

## Abstract

Ischemic heart diseases remain a challenge for clinicians. QiShenYiQi pills® (QSYQ) has been reported to be curative during coronary heart diseases with modulation of energy metabolism as one of the underlying mechanisms. In this study, we detected the effect of QSYQ and its components on rat myocardial structure, mitochondrial respiratory chain complexes activity and energy metabolism, and heart function after 30 min of cardiac ischemia, with focusing on the contribution of each component to its potential to regulate energy metabolism. Results showed that treatment with QSYQ and all its five components protected myocardial structure from damage by ischemia. QSYQ also attenuated release of myocardial cTnI, and restored the production of ATP after cardiac ischemia. AS-IV and Rb1, but not Rg1, R1, and DLA, had similar effect as QSYQ in regulation of energy metabolism. These results indicate that QSYQ may prevent ischemia-induced cardiac injury via regulation of energy metabolism, to which each of its components contributes differently.

## Introduction

Ischemic heart disease remains to be one of the diseases with highest mortality in the world (Yellon and Hausenloy, 2007). Primary percutaneous coronary intervention (PCI) is currently the most effective strategy to improve the clinical outcome for patients presenting acute myocardial infarction. Although PCI restores blood flow in myocardium, it frequently results in reperfusion injury and adds hazards to cardiac function via ROS generation, apoptosis, etc. (Ambrosio et al., 1986; Arslan et al., 2010).

During cardiac ischemia, hypoxia leads to uncoupling of oxidative phosphorylation of the respiratory chain, resulting in depletion of ATP (Wheaton and Chandel, 2011), which plays a key role in ischemic myocardial injury. ATP synthase, the main resource of ATP production, is a multisubunit complex consisting of 15 subunits including ATP 5D in mammals (Nesci et al., 2016). The activity of cardiac ATP synthase is reported to decrease in response to heart ischemia, followed by ATP depletion (Tu et al., 2013). Reduction of ATP leads to depolymerization of F-actin (Korn et al., 1987) and phosphorylation of myosin light chain (MLC), one of the major component of heart contraction machinery, and thus the abnormality of cardiac structure and function (Han and Ogut, 2010). Most studies on the management of I/R focus on the alleviation of oxidative stress, apoptosis, or inflammation occurred in reperfusion phase, while few strategy is available targeting energy metabolism disorder initiated in ischemia phase. Given the critical role of energy supply in minimizing ischemia injury, a regime able to protect energy metabolism dysfunction is needed, particularly for those patients who cannot access timely to a PCI treatment.

**Figure 1:**
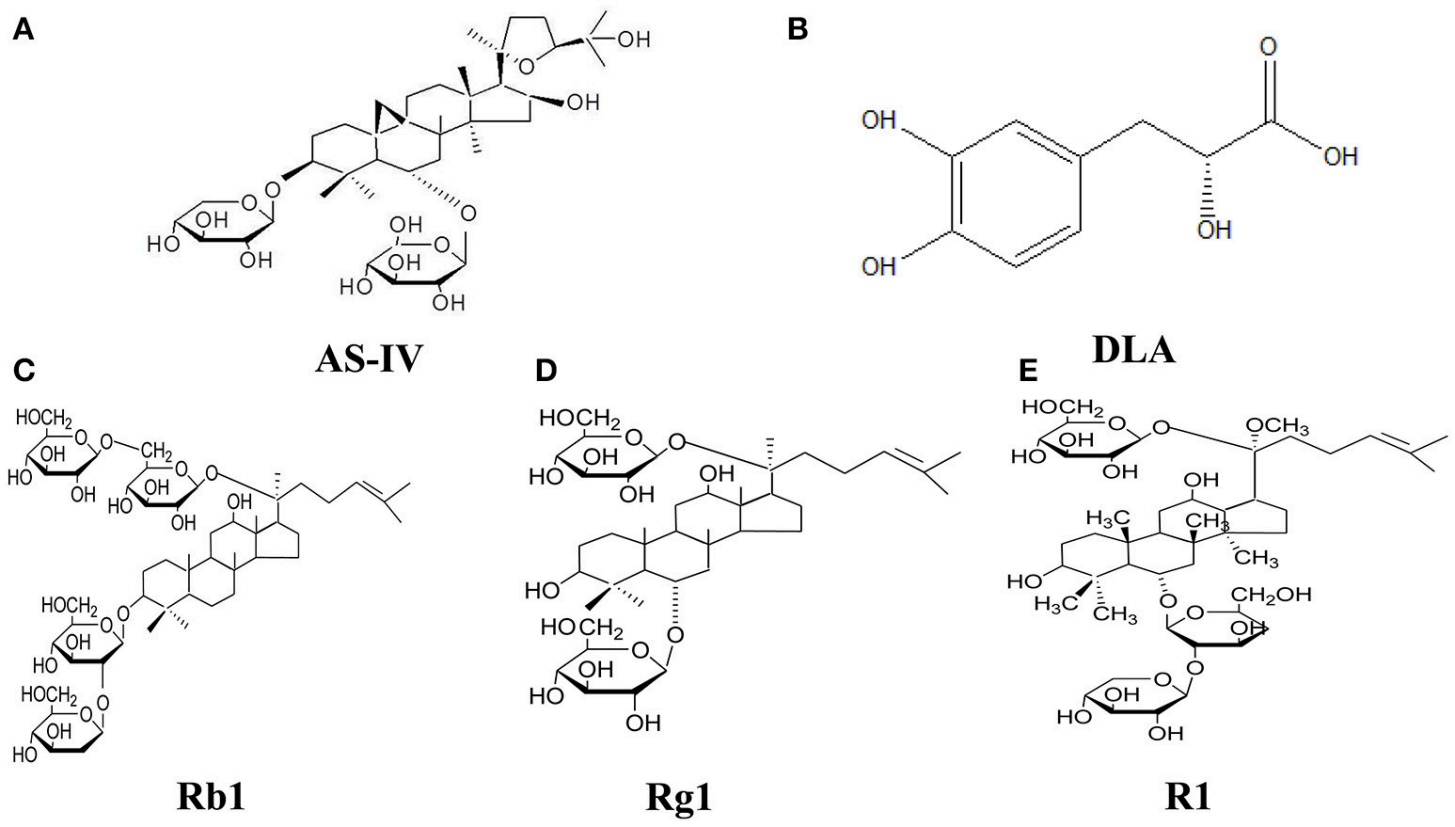
The chemical structure of components of QSYQ. **(A–E)** AS-IV, DLA, Rb1, Rg1, and R1.

QiShenYiQi Pills® (QSYQ) is a compound Chinese medicine composing of Radix Astragali (RA), Salvia miltiorrhiza (SM), Panax notoginseng (PN), and rosewood, which has been approved for treatment of coronary heart disease and angina by the Chinese State Food and Drug Administration. The efficiency of QSYQ in treatment of angina was documented and reviewed recently (Cao et al., 2017). Our previous study reported that QSYQ has the ability to attenuate energy metabolism deficiency and upregulate ATP 5D in cardiac I/R injury (Lin et al., 2013). QSYQ was also reported to be able to attenuate cardiac hypertrophy in a multicomponent and multitarget manner (Tang et al., 2013; Chen et al., 2015). The major active ingredients of QSYQ have been identified with the chemical structures showing in Figure 1. Of them, AS-IV (from RA) and Rb1 (from SM) are known as strong metabolism regulators, enhancing ATP production during cardiac I/R injury (Cui et al., 2017), R1 (from PN) has been proven having anti-oxidative (Wang et al., 2016) and anti-inflammatory effects (Su et al., 2016), as well as improvident role of energy metabolism (He et al., 2014), and Rg1 (from SM) exhibits anti-inflammation effects (Zhu et al., 2017). DLA has shown the potential of attenuating energy metabolism disorder (Yang et al., 2015) and antioxidant effect in cardiovascular protection (Wu et al., 2009) and vasodilatation (Lam et al., 2007). However, the contribution of each component in QSYQ to its effect on cardiac energy metabolism, especially the function of mitochondrial electron transport chain, remains unclear.

In this study, we explored the effect of QSYQ and its five main components on ischemic cardiac injury intending to gain insight into the role of each component in attenuating cardiac energy metabolism and myocardial structure during heart ischemia.

## Materials and methods

### Animals

Male Sprague-Dawley (SD) rats weighing 230–270 g were obtained from the Animal Center of Peking University [certificate no. SCXK (Jing) 2006-0008]. The rats were kept at 22 ± 2°C and relative humidity 40 ± 5%, being allowed access to standard diet and water. The investigations complied with the Guide of Peking University Animal Research Committee. All experimental procedures were approved by Peking University Biomedical Ethics Committee Experimental Animal Ethics Branch (LA2010-001).

### Regents

QiShenYiQi Pills® (Batch number: 20091005) was acquired from Tasly Pharmaceutical Co. Ltd. (Tianjin, China), the production process of which was in accordance with the guidelines of Good Manufacturing Practice and Good Laboratory Practice. The major components of QSYQ were determined by HPLC finger print. AS-IV, Rb1, Rg1, R1, and DLA were purchased from Feng Shan Jian Medicine Research Co. Ltd. (Kunming, Yunnan, China). QSYQ Pills and AS-IV were dispersed in saline before experiment to make a suspension in a concentration of 0.15 g/ml and 2.5 mg/ml, respectively, and Rb1, Rg1, R1, and DLA were dissolved in saline before experiment to make a solution in a concentration of 1.25 mg/ml.

### Cardiac ischemia model and experiment protocols

Animals were anesthetized with 2% pentobarbital sodium (60 mg/kg) by intraperitoneal injection, and placed in a dorsal position. A tracheal cannula was inserted via mouth with one end connected to an animal breathing apparatus (ALC-V8; Shanghai Alcott Biotech Co., Shanghai, China), which was set at the breathing ratio 1:1, the frequency 75 times/min, and tidal volume 12 ml/kg. A thoracotomy was carried out to expose the heart, and the left anterior descending coronary artery was ligated with a 5/0 silk for 30 min. Thirty min before ischemia, the animals in Rb1+I, Rg1+I, R1+I, DLA+I groups received Rb1, or Rg1, R1, DLA by continuous infusion through femoral vein at 5 mg/kg/h. Animals in S+Sham group and S+I group received saline in the same way at 5 ml/kg/h. Animals in AS-IV+I group and QSYQ+I group were administrated through gavage with AS-IV in saline at a dose of 10 mg/kg and QSYQ in saline at a dose of 0.6 mg/kg, respectively, 90 min before ischemia. And animals in QSYQ+Sham, AS-IV+Sham, Rb1+Sham, Rg1+Sham, R1+Sham, and DLA+Sham groups were treated following the same procedure except for suture silk ligation. The doses used for different drugs were determined based on our former studies (Lin et al., 2013; Tu et al., 2013; He et al., 2014; Yang et al., 2015; Cui et al., 2017; Li et al., 2018).

### Histological evaluation of myocardial tissues

At the end of ischemia, hearts were cut from the middle one third between the apex and the ligation point and the lower part was fixed in 10% formalin, then prepared for paraffin sectioning. The paraffin sections (5 μm) were stained with hematoxylin and eosin (HE) and observed and photographed by a microscope equipped with a digital camera (BX512DP70, Olympus) (Lin et al., 2013).

### Rhodamine phalloidine staining

Rat heart was perfused with saline followed by removal and fixation in 4% paraformaldehyde solution for 48 h. To reveal F-actin, heart paraffin section (5 μm) was prepared and subjected to staining with rhodamine phalloidine (R415; Invitrogen, Carlsbad, CA, USA) in accordance with the instruction of the manufacture. Nuclei were labeled with Hoechest 33342. A laser scanning confocal microscope (TCS SP5; Leica, Mannheim, Germany) was applied for observation of ischemic area at 40X magnification of objective (Lin et al., 2013).

### Ultrastructure examination

Rat hearts were perfused for 40 min with 4% paraformaldehyde and 2% glutaraldehyde (TedPella, Redding, CA, USA) in 0.1 mol/L phosphate buffer at a speed of 3 ml/min, and then removed. Myocardial tissue was collected at one third above the apex cordis, and left ventricle region was cut into 1 mm^3^ blocks. The tissue blocks were then fixed overnight at 4°C with 3% glutaraldehyde, washed three times with 0.1 mol/L phosphate-buffered solution, and post-fixed with 1% osmium tetraoxide for 2 h. The ultrathin sections were prepared, observed, and photographed with a transmission electron microscope (JEM 1230, JEOL, Tokyo, Japan) (Lin et al., 2013).

### Protein extraction

Rat hearts were removed after animals were perfused with saline under anesthesia. The left ventricle tissue sample was taken at 2 mm under ligature site, froze in liquid nitrogen, and stored at −80°C before use. Samples were thawed and centrifuged at 20,000 g, then total protein of tissue was extracted using total protein extraction kit (Applygen Technologies, Beijing, China), according to manufacturer's instruction (Cui et al., 2017).

### Western blot

The total protein concentration of each sample was determined twice with BCA protein assay kit (Applygen Technologies, Beijing, China) in accordance with the manufacture's instruction, taking the average as the concentration. The protein was then mixed with 5X electrophoresis buffer, separated in 10% SDS-PAGE, and transferred to polyvinylidene difluoride membrane. Membranes were blocked with 3% skimmed milk powder, rinsed with TBS-Tween for three times, 5 min each, then cut according to molecular weight and incubated at 4°C overnight with primary antibodies against glyceraldehyde-3-phosphatedehydrogenase (GAPDH) and cTnI (1:1,000, Abcam, Cambrige, MA, USA), and ATP5D (1:200, Santa Cruz, California, USA), respectively. After being rinsed three times, 5 min each, membranes were incubated with secondary antibody for 1 h at room temperature, and then rinsed again with TBS-Tween three times, 10 min each. The quantification of target protein was carried out by scanning densitometry in the X-film using a bio-image analysis system (Image-Pro plus 6.0; Media Cybernetic, Bethesda, MD, USA) (Cui et al., 2017).

### ATP, ADP, AMP content in myocardium

ATP, ADP, AMP content in myocardium was determined with ELISA kits (MULTISKAN MK3, Thermo, San Jose, CA, USA) using a microplate reader (MULTISKAN MK3, Thermo, San Jose, CA, USA) in accordance with the manufacturer's instructions (Cui et al., 2017).

### cTnI content in plasma

Blood was collected at the end of ischemia and plasma was prepared using heparin as an anticoagulant. The supernatant was collected after centrifugation for 15 min at 1,000 g. Determination of cTnI content was performed using rat cTnI ELISA Kit by microplate reader (MULTISKAN MK3; Thermo, San Jose, CA, USA) (Cui et al., 2017).

### Mitochondrial complex I, II, IV, V activity

Myocardium total protein was adjusted to a concentration of 5.5 mg/ml. The sample was added with detergent at a ratio 1/10 (v/v), mixed and incubated on ice for 30 min. The supernatant was collected after centrifugation at 12,000 g for 20 min. Complex I, II, IV, V activity was determined using mitochondria complex activity microplate assay kit (Abcam, Cambridge, UK), with setting the plate in the MULTISKAN MK3 enzyme micro-plate reader (Thermo Fisher Scientific Inc., Illinois, USA). The absorbance of each well was measured at 340 nm, 30°C for 60 min using a kinetic program. Mitochondrial complex activity was expressed as change in absorbance per minute at 340 nm (mOD/min) (Cui et al., 2017).

### Heart function test

Heart function was evaluated 30 min after ischemia by a bio-function experiment system BL-420F (Chengdu Taimen Technology Ltd., Chengdu, Sichuan, China), which was connected to a cannulation inserting into left ventricle through right carotid artery, and LVSP, LVDP, +dp/dtmax, and –dp/dtmax were assessed (Lin et al., 2013).

### Statistical analysis

All data were expressed as mean ± S.E.M. Statistical analysis was performed using one-way ANOVA followed by Newman–Keuls test. Data were analyzed using GraphPad Prism 5 software (GraphPad software Inc., USA). A *p-*value < 0.05 was regarded to be statistically significant.

## Results

### Effect of QSYQ and its components on myocardium histology of rat subjected to ischemia

**Figure 2:**
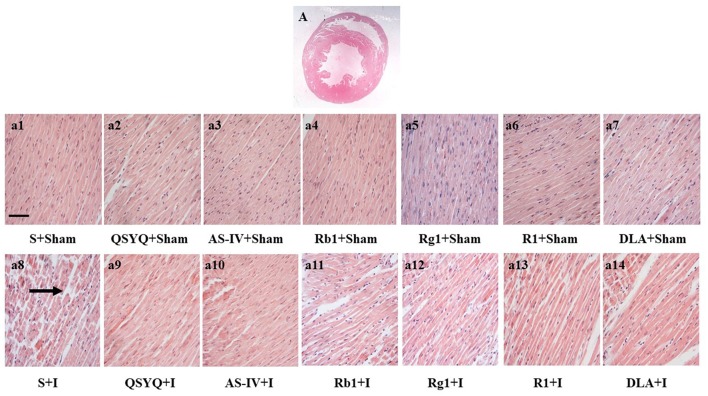
Effect of QSYQ and its components on myocardial morphology after ischemic challenge. **(A)** Entire section of heart tissue under HE staining. **(a1–a14)** Representative photographs of myocardium stained by HE in different groups at high magnification (bar = 100 μm). Arrow: ruptured myocardial fiber, *n* = 3.

The histology of myocardium stained by HE in different groups was examined with the results presenting in Figure 2. In S+I group, considerable morphological changes were observed in ischemic left ventricle areas of heart tissues compared with S+Sham group, including interstitium edema and disruption of myocardial fibers. However, QSYQ as well as all its five components remarkably prevented myocardial alterations after ischemia, with the best effect being found for QSYQ.

### Effect of QSYQ and its components on F-actin structure

**Figure 3:**
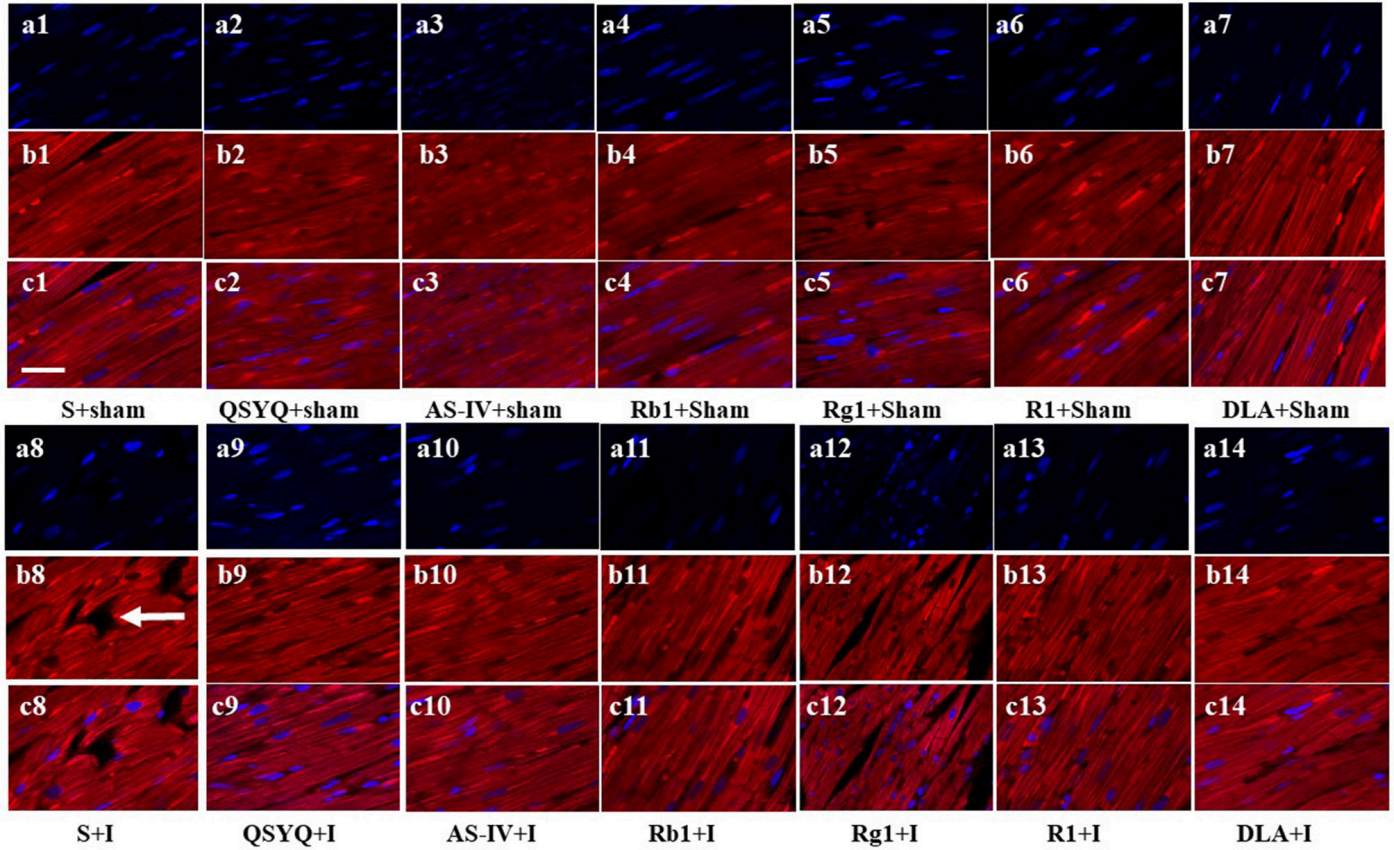
Effect of QSYQ and its components on F-actin structure. The representative photographs of rat myocardium with staining for F-actin **(c1–c14)**. Nuclei are stained with blue **(a1–a14)** and F-actin red **(b1–b14)**. Arrow: ruptured F-actin. Bar = 50 μm, *n* = 3.

Rhodamine phalloidine staining was applied to label F-actin and assess the alteration in myocardium structure. As presented in Figure 3, myocardium tissues in S+Sham groups displayed continuous F-actin bundles, while S+I group showed severe rupture in F-actin bundles, representing the damaged cardiac myofibrils. Obviously, ischemia-induced F-actin disruption was attenuated by QSYQ and all its five components.

### Effect of QSYQ and its components on myocardial ultrastructure of rat subjected to ischemia

**Figure 4:**
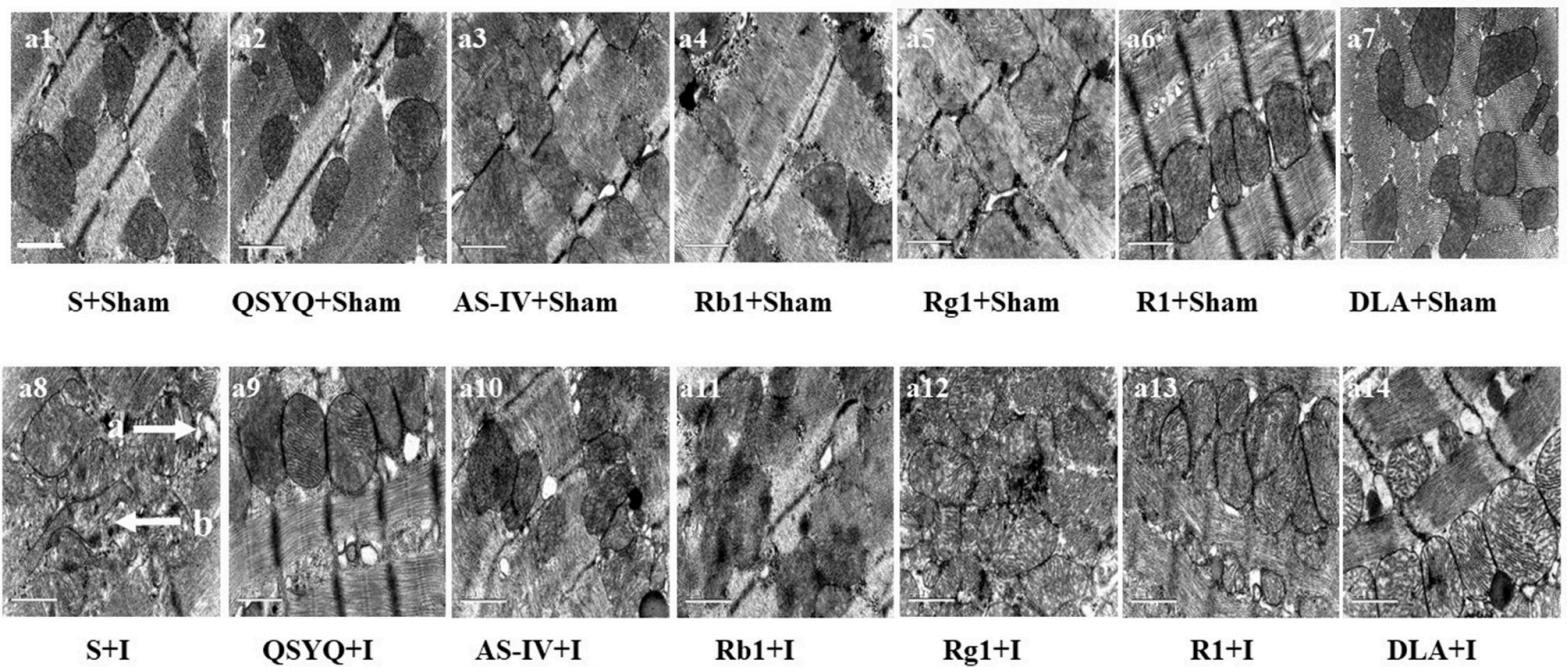
Effect of QSYQ and its components on myocardial morphology after ischemic challenge. **(a1–a14)** Presented are the representative electron micrographs of myocardium from different groups. Bar = 1 μm. a: disrupted myofibril, b: swelling mitochondria. *n* = 3.

Representative ultrastructural images of myocardial tissue in all groups are presented in Figure 4. As expected, myocardial tissue in S+Sham group revealed normal ultrastructure presenting regularly arranged myofibrils, well-preserved sarcomeres, and mitochondria. Ischemic challenge induced obvious myocardium ultrastructure injury, including disrupted myofibrils and swelling mitochondria, which were protected by QSYQ and its five components to different extent.

### Effect of QSYQ and its components on ATP 5D expression in myocardium and plasma of rats subjected to ischemia

**Figure 5:**
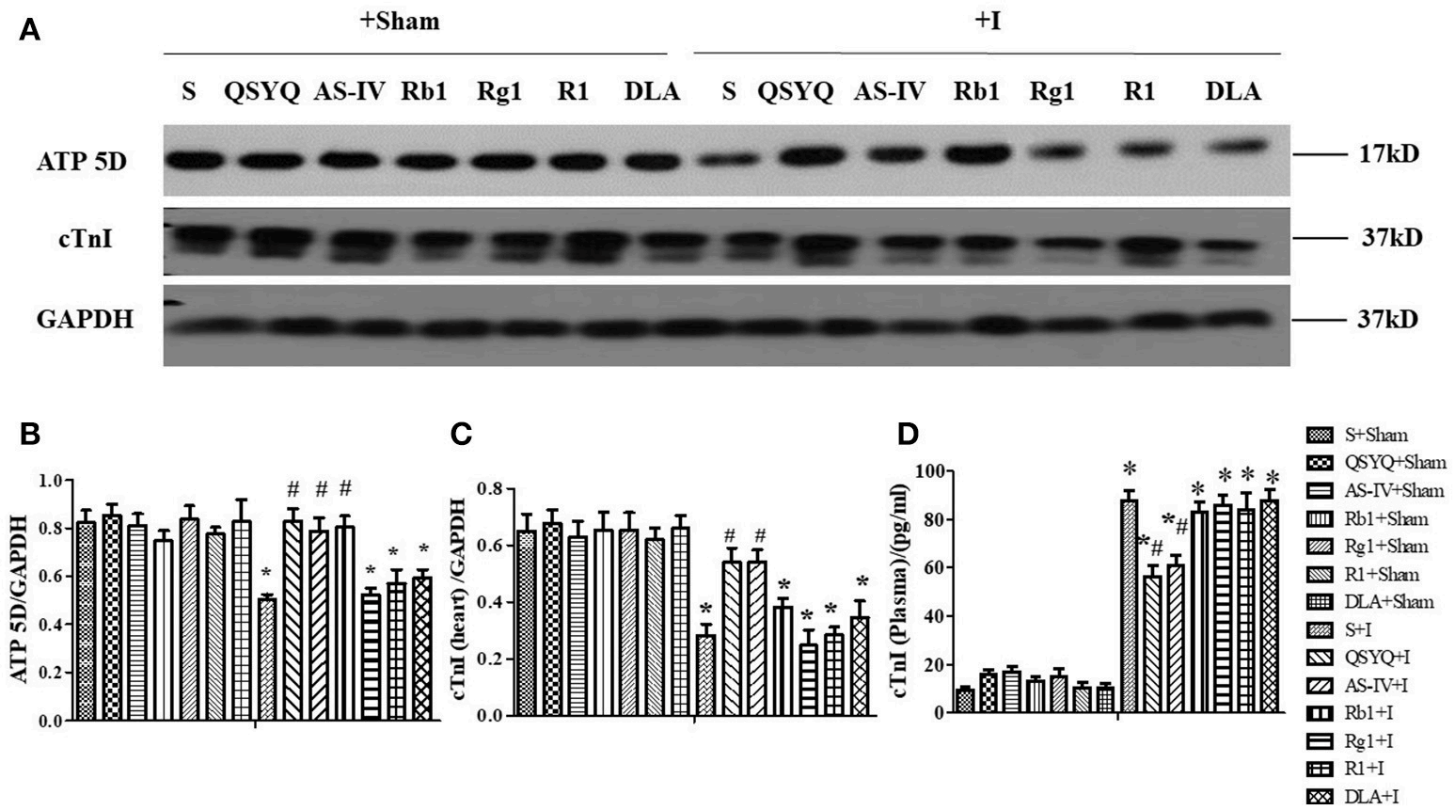
Effect of QSYQ and its components on ATP 5D in rat myocardium and cTnI level in rat myocardium and plasma subjected to ischemia. **(A)** The representative Western blot bands of ATP 5D and cTnI in myocardium in different groups. GAPDH was used as a loading control. *n* = 4. **(B,C)** The semi-quantitative analysis of ATP 5D and cTnI, respectively. **(D)** cTnI level tested by ELISA in plasma after ischemia. Data are expressed as the means ± S.E.M. *n* = 6. ^*^p < 0.05 vs. S+Sham group, ^#^*p* < 0.05 vs. S+I group.

We then determined the protein expression of ATP 5D, one subunit of ATP synthase, in cardiac tissues from different groups. As shown in Figures 5A,B, ATP 5D protein expression decreased significantly after cardiac ischemia, in comparison with S+Sham group, which was prevented by treatment with QSYQ, AS-IV, and Rb1, but not Rg1, R1 or DLA.

As a marker of myocardial damage, the level of cardiac troponin I (cTnI) in myocardium and plasma was determined using western blot and ELISA, respectively. Compared with S+Sham group, the level of cTnI in myocardium decreased significantly in response to ischemic challenge, as shown in Figures 5A,C. In contrast, the level of cTnI in plasma was very low in S+Sham group, but elevated impressively after ischemia challenge, as shown in Figure 5D. Of notice, ischemia-elicited change in cTnI level in both myocardium and plasma was significantly protected by treatment of QSYQ and AS-IV, but not Rb1, Rg1, R1, or DLA.

### Effect of QSYQ and its components on ATP production and activity of mitochondrial complexes in rat myocardium after ischemia

**Figure 6:**
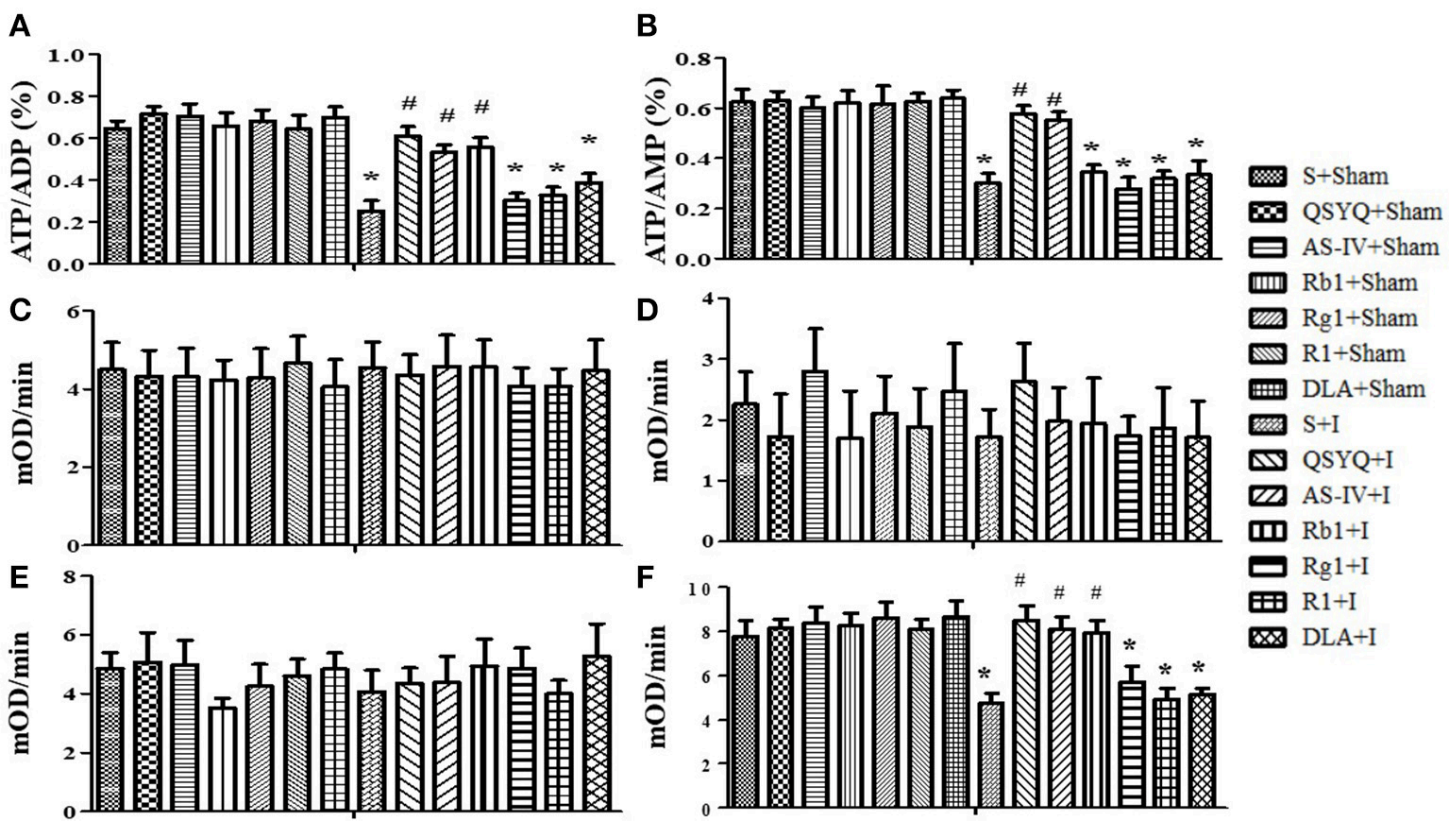
Effect of QSYQ and its components on ATP production and activity of mitochondrial complexes in rat myocardium subjected to ischemia. **(A,B)** The effects of QSYQ and its components on the ratio of ATP/ADP **(A)** and ATP/AMP **(B)** in myocardium in different groups. **(C–F)** The Effect of QSYQ and its components on the activity of mitochondrial complex I **(C)**, II **(D)**, IV **(E)**, and V **(F)** in myocardial tissue in various groups. All Data are expressed as the means ± S.E.M. *n* = 6. ^*^p < 0.05 vs. S+Sham group, ^#^*p* < 0.05 vs. S+I group.

To assess energy metabolism, the ratios ATP/ADP and ATP/AMP in cardiac tissue were determined in different conditions (Figures 6A,B). As compared with S+Sham group, ischemic challenge decreased ATP/ADP and ATP/AMP considerably, which indicates a disorder in energy metabolism balance. Treatment with QSYQ, AS-IV, and Rb1 but not Rg1, R1, or DLA significantly prevented ATP/ADP from decreasing by cardiac ischemia. A similar effect of these treatments was found on the ratio of ATP/AMP with an exception that Rb1 was ineffective in this case.

Then we evaluated activities of mitochondrial complexes I, II, IV, and V in cardiac tissues by ELISA. As noticed in Figures 6C–F, activities of complexes I, II, IV remained unchanged after ischemia for 30 min, and no effect of each treatment was observed on them. In contrast, activity of complex V diminished significantly after ischemia comparing to S+Sham group, which was considerably attenuated by QSYQ, AS-IV, and Rb1. On the other hand, Rg1, R1, and DLA did not affect the activity of complex V.

### Effect of QSYQ and its components on heart function in rats subjected to myocardium ischemia

**Figure 7:**
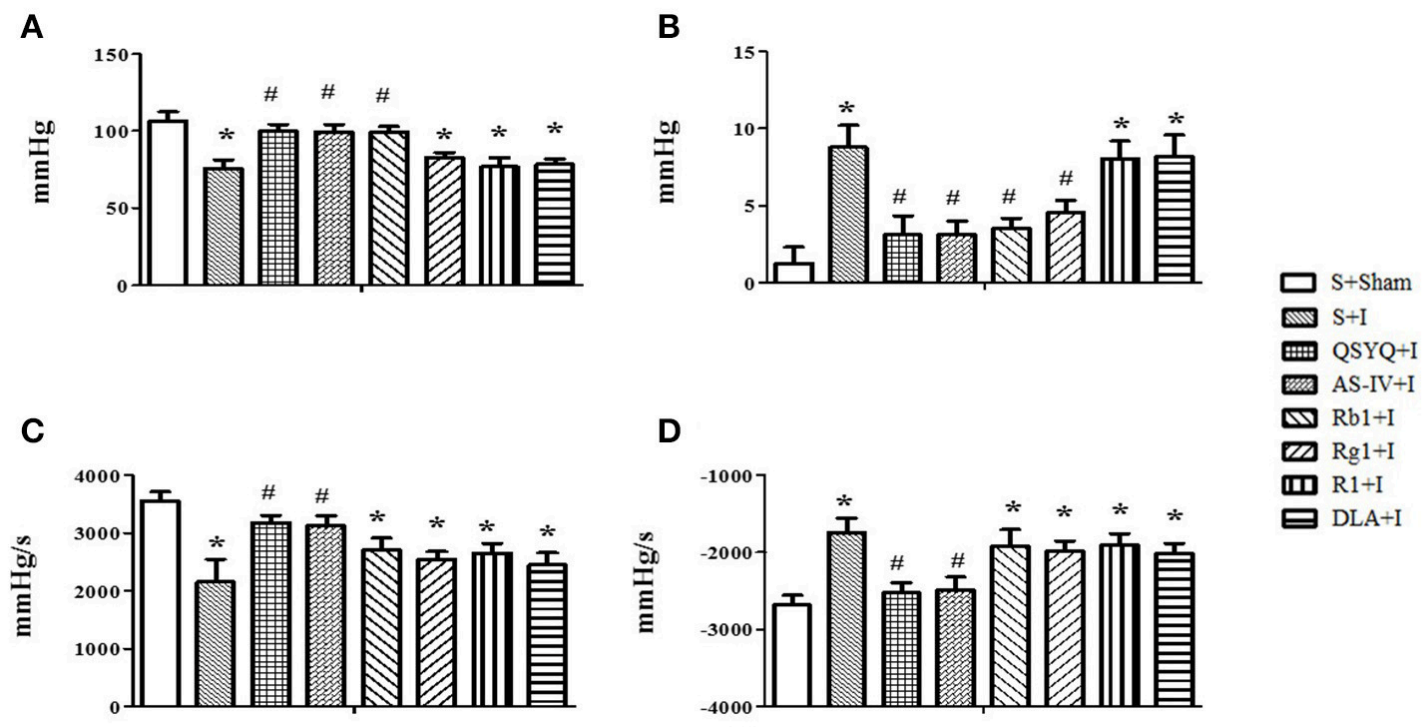
Effect of QSYQ and its components on heart function after ischemia. **(A–D)** Effect of QSYQ and its components on LVSP **(A)**, LVDP **(B)**, +dp/dtmax **(C)**, and –dp/dtmax **(D)** in different groups, respectively. All Data are expressed as the means ± S.E.M. *n* = 6. ^*^p < 0.05 vs. S+Sham group, ^#^*p* < 0.05 vs. S+I group.

Heart function was evaluated to test the role of QSYQ and its components in preventing heart dysfunction. As noticed in Figures 7A–D, in comparison with S+Sham group, ischemia challenge caused a significant decrease in LVSP and +dp/dt, and increase in LVDP and –dp/dt, indicating an impairment of heart function. QSYQ and AS-IV alone attenuated all these alterations caused by ischemia challenge, while Rb1 only alleviated the change in LVSP and LVDP, and Rg1 only alleviated LVDP. No effect was observed for R1 and DLA on these parameters.

## Discussion

In this study, we found that QSYQ significantly inhibited ischemia-induced rat myocardial injury, as demonstrated by the improvement of myocardial morphology and heart function and decline in cardiac cTnI release. QSYQ also relieved myocardial energy disorders manifested by the increase in ATP production, ATP 5D protein expression, and ATP synthase activity. Furthermore, we provided evidence showing that all the five components of QSYQ attenuated myocardial morphology induced by cardiac ischemia. Among them, AS-IV and Rb1 had alleviating effect on ATP production, ATP 5D expression, ATP synthase activity, and heart function during cardiac ischemic injury. Rg1 also attenuated impaired heart function caused by ischemic challenge. These findings suggest QSYQ as a potential alternative treatment for patients presenting with ischemic heart diseases like acute angina pectoris with modulation of energy metabolism as one of the major underlying mechanisms, to which AS-IV and Rb1 are the main contributors.

Energy metabolism disorder and resultant ATP deficiency occurs in ischemia- injured myocardium, which is responsible for a diversity of insults, including cation pumps dysfunction, and calcium overload, resulting in damage to the cell function and structure. In addition, the hydrolysis products of ATP are ADP and AMP, and rising of AMP/ATP and ADP/ATP ratios leads to activation of AMPK, which triggers a variety of signaling pathways concerning energy metabolism (Ke et al., 2017; Lin and Hardie, 2018). The immediate consequence of ischemia is the hypoxia and deprivation of nutrition, which impairs the respiratory chain in mitochondria. Respiratory chain consists of five complexes, a damage to any of which may cause interruption of the oxidative phosphorylation reaction. The present study revealed that ischemia for 30 min led to a downregulated expression of ATP 5D and ATP synthase activity, the fifth complex of mitochondrial respiratory chain, while had no effect on mitochondrial complex I, II, and IV, implying ATP5D dysfunction as the cause for the observed deficiency of ATP. The exact reason for the impairment of ATP 5D is at present unknown. Considering the time frame of the challenge, this impairment is unlikely caused by dysfunction of transcription, but possibly due to disordered translation or post translational modification. As expected, in the present study, QSYQ was observed to protect ATP5D and complex V in myocardium from decrease in expression and activity after ischemia. Among the ingredients in QSYQ, AS-IV, and Rb1 exhibited a similar potential as QSYQ in protection of ATP5D and complex V, consistent with the previous reports (He et al., 2014; Jiang et al., 2017), suggesting these two ingredients as the main contributors to the role of QSYQ in attenuation of energy metabolism in the present condition.

On the other hand, R1 was not observed to affect the ATP5D expression and complex V activity in the present setting, a result that is in contrast to a previous finding (He et al., 2014), suggesting that R1 targets at a link of ATP5D impairment process different from that for AS-IV and Rb1, and this inconsistency may be accounted for by the difference in the experiment protocols. In the present study, the challenge was 30 min ischemia while in the previous study was 30 min ischemia followed by reperfusion. It is likely that ATP5D expression impaired in both conditions, though, but at different link in different condition.

We have previously reported that DLA, another major ingredient of QSYQ, is able to upregulate ATP production in a cardiac ischemia and reperfusion model. In the present study, DLA did not show any effect on the decreased ATP production after 30 min of myocardium ischemia. This result is predicable considering the fact that in the previous study DLA was found to upregulate ATP production by binding to Sirt1 thus relieve the function of complex I of respiratory chain, which was impaired after ischemia and reperfusion, while in the present study complex I kept unchanged in the expression and activity after 30 min of ischemia. Likewise, we did not find any protective effect of Rg1 on the reduced ATP production and impaired ATP5D expression and ATP synthase activity after 30 min ischemia. However, Rg1 was observed to improve the myocardium structure and cardiac function after ischemia, although to a less extent. This effect may be attributable to the beneficial role of Rg1 in the ischemia-caused insults other than energy metabolism disorder, such as inflammation (Yu et al., 2017).

Taken together, comparing the results of the present study with the previous reports, it is most likely that the energy metabolism impairs in the myocardium after ischemia or ischemia/reperfusion, and the cause for the impairment may vary depending on the condition. Most of the ingredients included in QSYQ have potential to attenuate energy metabolism disorder caused by ischemia and reperfusion, but with a distinct target for each ingredient to exert its action. As a result, not all the ingredients are at work to attenuate energy metabolism in any condition. This results highlight the necessity to include in QSYQ multiple ingredients effective to attenuate energy metabolism.

The present study revealed that ischemia for 30 min resulted in a significantly decrease of cTnI in myocardium and increase in plasma, suggesting the occurrence of cardiac injury. This injury was also evidenced by the findings in histology and ultrastructure of myocardium. The effect of QSYQ and each ingredient on the change of cTnI, ATP5D and ATP production suggested the importance of energy supply in protection of cardiomyocytes from ischemic injury. However, we failed to find the infarction in TTC-stained myocardium slices in the present study (Data not shown), which is possibly due the infarction being too small to be detected by TTC stain.

In summary, these results suggested that QSYQ could be adopted as potential alternative treatment for patients presenting with ischemic heart diseases like acute angina pectoris, especially for those who cannot access to PCI treatment timely. This potential of QSYQ is correlated with regulation of energy metabolism to which AS-IV and Rb1 are the main contributors.

## Author contributions

Y-CC: Performed the experiments, analyzed the data, and wrote the manuscript; LY, B-HH, and XC: Contributed to animal experiments; C-SP: Contributes to biochemical experiments; J-YF and J-YH: Revised the manuscript; J-YH: Designed and funded the research, interpreted the data, and finally approved the submission of this manuscript. All authors have read and agreed with the manuscript.

### Conflict of interest statement

The authors declare that the research was conducted in the absence of any commercial or financial relationships that could be construed as a potential conflict of interest.

## Abbreviations

AAR: Area At Risk
ADP: Adenosine Diphosphate
AMP: Adenosine Monophosphate
AMPK: Adenosine Monophosphate Kinase
AS-IV: Astragaloside IV
ATP: Adenosine Triphosphate
cTnI: Cardiac Troponin I
DLA: 3,4-dihydroxyphenyl lactic acid
ELISA: Enzyme-linked Immunosorbent Assay
HE: Hematoxylin/eosin
I/R: Ischemia/Reperfusion
LA: Left Ventricular Area
MDA: Malonidialdehyde
MRC: mitochondrial respiratory chain
NS: Natural Saline
PCI: percutaneous coronary intervention
QSYQ: QiShenYiQi Pills®
Rb1: Ginsenoside Rb1
Rg1: Ginsenoside Rg1
ROCK: Rho-associated Coiled-coil Containing Protein Kinase
ROS: Reactive Oxygen Species
R1: Notoginsenoside R1.
